# A graft inversion technique for retrograde type A aortic dissection after thoracic endovascular repair for type B aortic dissection

**DOI:** 10.1186/s13019-019-0851-9

**Published:** 2019-02-04

**Authors:** Wenbin Hu, Yiran Zhang, Lei Guo, Jingya Fan, Yuan Lu, Liang Ma

**Affiliations:** 1 Department of Cardiothoracic Surgery, The First Affiliated Hospital, School of Medicine, Zhejiang University, No.79 Qinqchun Road, Hangzhou, 310003 Zhejiang China; 2 Department of Cardiothoracic Surgery, Affiliated Hospital of Shaoxing University (The Shaoxing Municipal Hospital), Shaoxing, China

**Keywords:** Retrograde aortic dissection, Cardiac surgical procedures, Endovascular aortic repair, Type B aortic dissection

## Abstract

**Background:**

Retrograde type A aortic dissection (RTAD) is a rare but life-threatening complication after thoracic endovascular aortic repair (TEVAR) for type B aortic dissection (TBAD). A graft inversion technique was applied to distal anastomosis in total arch replacement for this complicated dissection. We reviewed our results of the processing for this serious complication. The aim is to evaluate the feasibility of this technology.

**Methods:**

From January 2013 to December 2017, 20 patients (80% male, mean age 50.9 ± 9.5 years) with retrograde type A aortic dissection after thoracic endovascular aortic repair for type B aortic dissection were scheduled for surgical treatment at our center. All patients underwent an ascending aorta and total aortic arch replacement procedure. The 20 patients were divided into two groups, 1 group involved 9 patients underwent surgery using stepwise technique; the graft inversion technique was performed in the other group containing the remaining 11 patients. The postoperative variables, including cardiopulmonary bypass time, the circulatory arrest time, the aortic cross clamp time, were analyzed. Meanwhile we also analyzed the postoperative mortality and complications to evaluate the early and mid-term outcomes of surgical treatment for RTAD after TEVAR.

**Results:**

In-hospital mortality was 10% (2 of 20 patients). No patient developed postoperative paraplegia, renal failure, stroke, or distal anastomotic bleeding. Two patients developed renal insufficiency, one developed neurologic insufficiency, and one developed pulmonary infection, all of which were managed accordingly. Cardiopulmonary bypass (CPB) time, and circulatory arrest time were significantly shorter in the graft inversion group than in the stepwise group (165.8 ± 37.9 min versus 206.1 ± 46.8 min, *p<*0.05; 34.5 ± 5.6 min versus 42.4 ± 9.5 min, *p<*0.05, respectively). The 18 survivors had a mean follow-up of 25.8 ± 18.2 months, and all patients remained alive and well.

**Conclusion:**

Graft inversion can enable a secure distal anastomosis under good surgical exposure, resulting in reduced durations of CPB, and circulatory arrest for RTAD after TEVAR. Surgical treatment could be a safe alternative for treatment of this patients.

**Electronic supplementary material:**

The online version of this article (10.1186/s13019-019-0851-9) contains supplementary material, which is available to authorized users.

## Introduction

Dake [1] and Nienaber [2] first applied thoracic endovascular aortic repair (TEVAR) for thoracic aortic disease in 1999; this technique has since been increasingly used as a safe and less-invasive treatment option for patients with Stanford type B aortic dissection (TBAD). However, TEVAR may lead to some potentially fatal complications. One of the most serious complications is retrograde type A aortic dissection (RTAD), which has a low incidence but a high mortality rate.

In the process of total arch replacement for type A aortic dissection, sometimes distal anastomosis is difficult, and bleeding from it is a tricky problem because of limited surgical exposure. Therefore, surgeons are seeking better technique for distal anastomosis. Koyu Tanaka et al. [3] modified a new procedure, graft inversion technique, which was applied to distal anastomosis in a total arch replacement for thoracic aortic aneurysm. In this paper, we report this technique which first applied to the treatment of RTAD after TEVAR, and summarized our experiences in surgical treatment for this serious complication.

## Patients and methods

### Patients

**Fig. 1:**
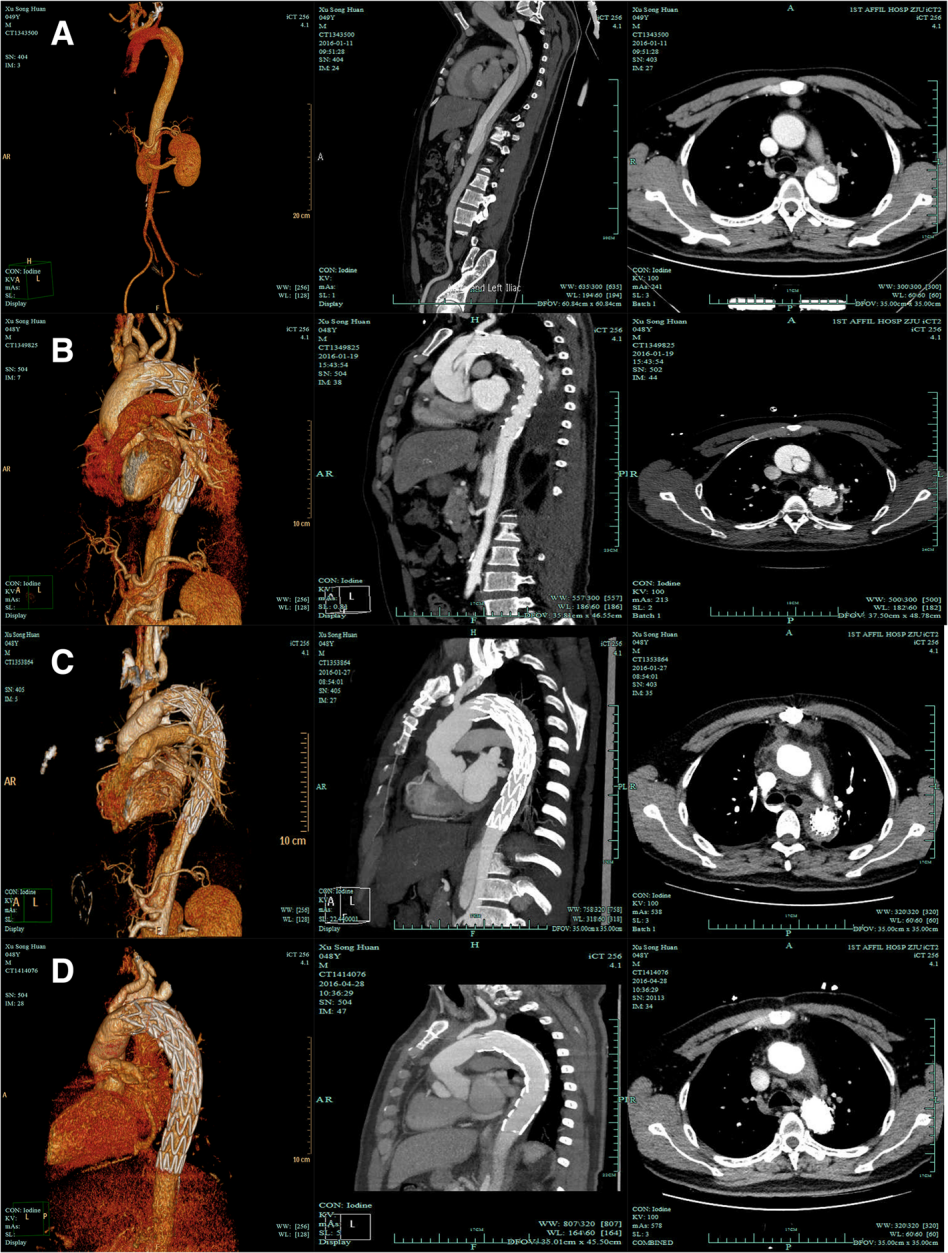
Computed tomography angiography (CTA) of a patient with RTAD related to TEVAR. **a** preoperatively, CTA showing type B dissection. **b** CTA reveals dissection of the ascending aorta 8 days after TEVAR. **c** The true lumen of the ascending aorta returned to normal after surgery. (D) Follow-up of CTA at 3 months, the false lumen thrombosis

Between January 2013 and December 2017, 571 patients with TBAD underwent TEVAR at our center. Patients with connective tissue disorders such as Marfan were excluded from consideration for TEVAR. Twelve (2.1%, 12 of 571) patients suffered RTAD after TEVAR at our center, and eight had been transferred to our center from other hospitals for the same reason. In all patients, the diagnosis was confirmed with computed tomography angiography (CTA). Figure 1 presents the CTA images of a patient who developed RTAD after TEVAR. Sixteen patients were diagnosed within 14 days after TEVAR (80%). The time interval of the other four patients from TEVAR to RTAD ranged from 27 days to 11 years. The group had a mean age of 50.9 ± 9.5 years and included 16 men (80%) and 4 women (20%). Sixteen patients had hypertension (80%), six had Marfan syndrome which were all transferred from other hospitals (30%), one had coronary artery disease (5%), one had renal insufficiency (5%), and one had diabetes (5%). Six additional cases involved aortic valve insufficiency. Most patients (75%) experienced a new onset of chest pain or back pain, while two (10%) complained of headache or syncope. The characteristics of the 20 patients are shown in Table 1. The study was approved by the Institutional Review Board of The First Affiliated Hospital, School of Medicine, Zhejiang University.

**Table 1:** Patient characteristics

Characteristic	All Patients (*n* = 20)
Age (years)	40–72 (50.9 ± 9.5)
Male	16 (80%)
Female	4 (20%)
Preoperative comorbidity	
Hypertension	16 (80%)
Marfan syndrome	6 (30%)
Coronary artery disease	1 (5%)
Renal insufficiency	1 (5%)
Diabetes	1 (5%)
Aortic insufficiency	6 (20%)
Symptom	
Chest or back pain	15 (75%)
Headache	1 (5%)
Syncope	1 (5%)
Asymptomatic	2 (10%)
Landing zone within aortic arch	
Zone 1	2 (16.7%)
Zone 2	7 (58.3%)
Zone 3	3 (25%)
Onset of retrograde type A aortic dissection within 14 days after TEVAR	16 (80%)

Data are presented as n (%) or means (ranges)

Detailed stent graft information was unclear for the eight patients transferred from other hospitals. The following stent grafts were used in the 12 patients who underwent TEVAR at our center: Valiant (Medtronic Cardiovascular, Santa Rosa, CA) in six patients, TAG (W. L. Gore and Associates, Flagstaff, AZ) in one, Zenith TX2 (Cook Inc., Bloomington, IN) in three, and Ankura (Lifetech, Shenzhen, China) in two. Thoracic stent graft oversizing ranged from 7.4 to 13.33% (mean, 10.79% ± 1.70%). Table 2 presents the details of the initial stent graft devices and oversizing.

**Table 2:** Initial stent graft devices used at our center

	Our center (*n* = 12)
Stent graft device	
Valiant (Medtronic)	6 (50%)
Gore TAG (Gore)	1 (8.3%)
Zenith TX2 (Cook)	3 (25%)
Ankura (Lifetech)	2 (16.7%)
Oversizing % (mean %)	7.4–13.33 (10.79 ± 1.70)

Data are presented as n (%) or means (ranges)

### Methods

Twenty patients, divided into two groups, underwent an ascending aorta and total aortic arch replacement procedure. 9 patients underwent surgery using stepwise technique; the graft inversion technique was performed on the remaining 11 patients. The variables, which included cardiopulmonary bypass time, the circulatory arrest time, the aortic cross clamp time, were analyzed. Meanwhile we also analyzed the postoperative mortality and complications to evaluate the early and mid-term results of surgical treatment for RTAD after TEVAR.

All collected clinical date were analyzed by SPSS 21.0, expressed as mean values ± standard deviation for continuous variables, and as percentages for categorical variables. *T*-tests were performed for all continuous variables in both groups. *P* values ≤0.05 were considered significant.

### Surgical technique

Median sternotomy was performed in all 20 cases. The right axillary artery was exposed routinely for cardiopulmonary bypass (CPB) and selective cerebral perfusion (SCP). An arterial cannula was inserted into the right axillary artery, a dual-stage caval cannula was inserted into the right atrium, and a left heart drainage tube was inserted into the right superior pulmonary vein. CPB flow was maintained at approximately 2.2 L/min, and patients were cooled to a rectal temperature of 28 °C. During this period, the ascending aorta brachiocephalic trunk was cross-clamped, the aorta was opened, and the heart was arrested with cold blood cardioplegia through the left and right coronary arteries. When aortic proximal heart lesions were detected, partial transaction of the aortic root replacement and aortic valve replacement or plasty were performed immediately. CPB was ceased once the rectal temperature was cooled to 22 °C, and the brain was perfused through the right axillary artery as soon as possible. The arteriae brachiocephalic trunk, left common carotid artery, and left subclavian artery were blocked in order. The aortic arch was then opened longitudinally, and the RTAD tear and endovascular stent graft were explored.

**Fig. 2:**
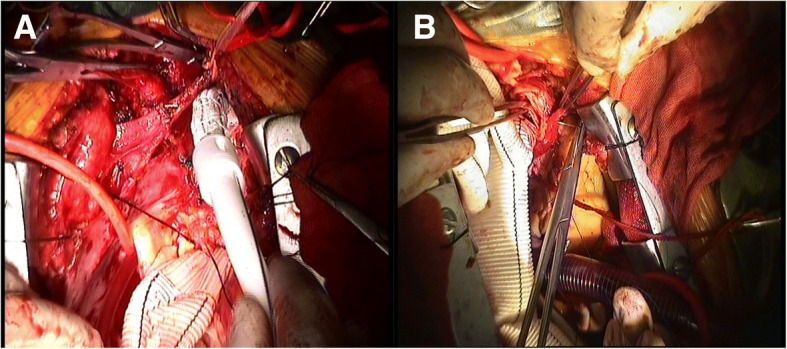
The stepwise technique of distal anastomosis: **a** Insertion of an elephant trunk into the true lumen of the descending aorta. **b** Suture of the descending aorta wall to a 4-branched prosthetic graft

**Fig. 3:**
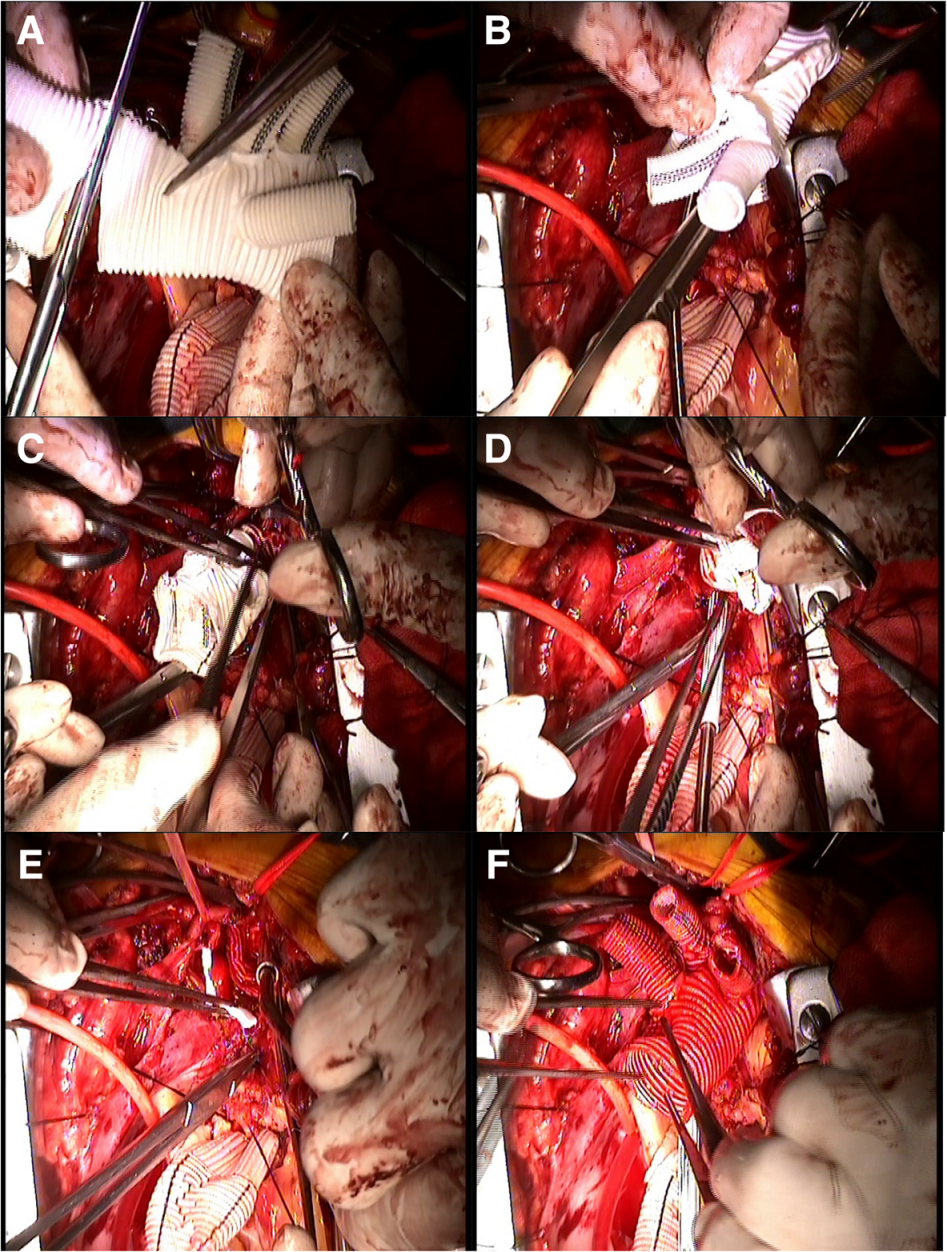
The graft inversion technique of distal anastomosis: **a** A 4-branched prosthetic graft cut to an appropriate length. **b** and **c** Careful inversion of the graft using forceps. **d** Forward insertion of the inverted graft into the true lumen of the descending aorta. **e** Distal anastomosis is performed using 4–0 polypropylene horizontal mattress sutures and lined with Dacron®. **f** The inverted graft was pulled up proximally after distal anastomosis was completed

To avoid a new intimal tear in the fragile aortic wall during endovascular stent removal, we retained all previous endovascular stent grafts and trimmed the bare wire springs from the proximal edges of endovascular grafts. In the first nine patients, an elephant trunk was inserted into the true lumen of the descending aorta through the opened aortic arch under direct vision. The proximal ends of the stent graft (after trimming) and Dacron®-lined descending aorta wall were then sewn together to a 4-branched prosthetic graft progressively (Fig. 2). Following technological refinements, an elephant trunk was no longer placed in the descending aorta of the latter 11 patients. Instead, graft inversion technique which we first applied to distal anastomosis. Our specific operation steps are as follows: (1) The 4-branched prosthetic graft is trimmed to a suitable length. (2) The quadrifurcated graft is completely inverted using forceps. (3) The inverted graft is carefully inserted into the descending aorta. (4) Distal anastomosis is performed using 4–0 polypropylene horizontal mattress sutures and lined with Dacron® under good surgical exposure. (5) Finally, the inverted graft is pulled up (Fig. 3 and Additional file 1: Figure S1). An additional video file shows the graft inversion technique in more detail (Additional file 2).

After completion of the distal anastomosis, the antegrade systemic perfusion resumed through the fourth branch of the graft. The left common carotid artery was then reconstructed. The left subclavian and innominate arteries were then anastomosed to the 4-branched graft in that order. Finally, the ascending aorta was anastomosed to the 4-branched graft.

## Results

Twelve patients who suffered RTAD after TEVAR at our center. The corresponding institutional incidence of RTAD was 2.1% (12 of 571). In 16 of the 20 patients, we observed new entry tears located at the distal convexity of the aortic arch near the bare metal springs at the proximal ends of the stent grafts when we explored the aortic arch in the operation. In the remaining four cases, the tears were located far from the bare metal springs, which excludes the stent graft as the cause.

The following procedures were performed: ascending aorta replacement, total arch replacement, and elephant trunk stent implantation in six cases; aortic valve repair, ascending aorta replacement, total arch replacement, and elephant trunk stent implantation in three cases; ascending aorta and total arch replacement in six cases; aortic valve replacement, ascending aorta replacement, and total arch replacement in three cases; and Bentall and total arch replacement in two cases. The mean CPB, aortic cross-clamp, and circulatory arrest times were 184.9 ± 46.7, 133.0 ± 34.8, and 38.0 ± 8.3 min, respectively. The mean durations of postoperative ventilator assistance, intensive care unit stay, and total hospitalization were 48.7 ± 31.3 h, 5.3 ± 2.5 days, and 14.1 ± 7.1 days, respectively. CPB time, and circulatory arrest time were significantly shorter in the graft inversion group than in the stepwise group (165.8 ± 37.9 min versus 206.1 ± 46.8 min, *p<*0.05; 34.5 ± 5.6 min versus 42.4 ± 9.5 min, *p<*0.05, respectively). No significant difference in aortic cross clamp time between the two groups (124.5 ± 32.1 min versus 142.8 ± 36.5 min, *p=*0.25). Relevant results are indicated in Tables 3 and 4.

**Table 3:** Surgical data

Variable	All Patients (n = 20)
Cardiopulmonary bypass time	184.9 ± 46.7 min
Aortic cross-clamp time	133 ± 34.8 min
Circulatory arrest time	38 ± 8.3 min
Ascending aorta + Total arch + SET	6 (30%)
AVP + Ascending aorta + Total arch + SET	3 (15%)
Ascending aorta + Total arch	6 (30%)
AVR + Ascending aorta + Total arch + SET	3 (15%)
Bentall + Total arch	2 (10%)

Data are presented as n (%) or means (ranges). *AVP* Aortic valve repair, *AVR* Aortic valve replacement, *SET* Stented elephant trunk

**Table 4:** Comparative perioperative parameters

Variables	Graft inversion (*n* = 11)	Stepwise (*n* = 9)	P
Cardiopulmonary bypass time	165.8 ± 37.9 min	206.1 ± 46.8 min	0.047
Aortic cross-clamp time	124.5 ± 32.1 min	142.8 ± 36.5 min	0.25
Circulatory arrest time	34.5 ± 5.6 min	42.4 ± 9.5 min	0.031

Data are presented as n (%) or means (ranges). All perioperative variables were analyzed by *t*-test. *P* ≤ 0.05 was regarded as statistic significance

Two patients died of multiple organ failure on the fifth and seventh postoperative days, for a mortality rate of 10%. During the early postoperative period, new-onset renal insufficiency occurred in two cases, transient cerebral disorder in one case, and pulmonary infection in one case. No patient developed spinal cord injury, stroke, or renal failure. Details of the complications are presented in Table 5.

**Table 5:** Early postoperative complications

Complication	All Patients (n = 20)
Death	2 (10%)
Renal insufficiency	2 (10%)
Transient cerebral disorder	1 (5%)
Pulmonary infection	1 (5%)

Data are presented as n (%) or means (ranges)

Eighteen survivors completed follow-up for a mean period of 25.8 ± 18.2 months. No patient died during the follow-up period, and no recurrences of aortic disease required reoperation. All patients recovered well and achieved satisfactory results and an improved quality of life.

## Discussion

TEVAR has become the first-choice therapy for patients with uncomplicated TBAD. However, RTAD, a catastrophic complication of this interventional procedure, has been extensively described. Although the incidence of RTAD was reported to be 1.3–4% [4, 5], the overall mortality rate was as high as 33.6–57% [6–9].

Given the evidence reported in the literature, RTAD may be associated with device which damage to the aortic wall during or after TEVAR, or as the outcome of natural progression of disease. The main possible risk factors for RTAD after TEVAR are as follows. (1) Direct injury occurs during the intervention. Aortic dissection alters the original shape of the aorta and causes intimal fragility, the resulting anatomic abnormality can trigger intimal damage in the easily injured aortic wall during guide wire manipulation, stent graft deployment, and balloon dilation. Subtle cardiac cycle–related movement of the semirigid stent graft also may cause RTAD during TEVAR. (2) The stent graft can be excessively oversized. According to international consensus, the degree of oversizing in a case of aortic dissection should be 15–20% to provide sufficient radial force to reduce the incidence of postoperative complications such as aneurysm neck dilatation, type I endoleak, and device migration [10–12]. However, Canaud et al. [13] found that each 1% increase in endograft oversizing beyond 9% increased the risk of RTAD by 14%, as the excessive radial force caused by aggressive oversizing could easily damage the fragile aortic wall. Lei et al. [14] observed that patients in whom stent graft oversizing remained at or below 5% experienced a lower incidence of RTAD with no significant increase in the stent migration and endoleak rates. Endovascular treatment for aortic dissection aims to plug the intimal tear, whereas the endograft provides only the necessary radial force to ensure complete attachment to the aortic wall. Therefore, excessive oversizing should be avoided, and an oversizing rate of 5% or less may be a suitable option for TEVAR of type B dissection. (3) Pathological changes in the aortic wall itself also comprise a high risk factor for RTAD after TEVAR. For example, patients with connective tissue disorders such as Marfan syndrome have a significantly increased incidence of RTAD. In a series of reoperations for complications of endovascular aortic repair, Spiliotopoulos and Colleagues [15] reported that 16 of 45 patients with a previous thoracic endograft had a connective tissue disorder (14 Marfan syndrome, 2 Loeys–Dietz syndrome), and all 16 developed aortic dissection. Accordingly, endovascular stent grafts should not be used routinely in patients with connective tissue disorders [16]. (4) A proximal landing zone diameter greater than 40 mm was also associated with an increased risk of RTAD [17]. (5) The timing of RTAD may be a factor because acute dissection and fragility appear to have a correlation with a higher incidence of RTAD after TEVAR [6].

Given the high mortality rate associated with RTAD, open surgery is necessary. In contrast to patients with a primary type A aortic dissection, the aortic arch requires exploration and resolution in patients with RTAD, and total arch replacement must be performed under deep hypothermic circulatory arrest. Therefore, great care should be taken to protect the brain and spinal cord. However, this operation is simultaneously complicated by the presence of previous endovascular stent grafts in the aortic cavity. Researchers have reported various methods addressing these previous grafts. (1) The previous endovascular stent grafts may be left in place, and the 4-branched prosthetic graft can be directly anastomosed to the aortic wall. (2) The previous endovascular stent grafts can be removed completely. (3) The bare waved wire of the endovascular stent graft proximal edge can be trimmed, and distal anastomosis can then be completed. In our 20 patients, we left the previous endovascular stent grafts in place to prevent a new initial tear in the fragile aortic wall during removal. However, we did trim the bare waved wires of the graft proximal edges.

We note that direct anastomosis between an artificial blood vessel and a distal aorta containing stent grafts is unreliable and can easily lead to anastomotic bleeding. Therefore, we deployed an elephant trunk into the descending aorta (stent-in-stent) along with the proximal ends of the stent graft and aortic wall, which were sewn together to a 4-branched prosthetic graft. This procedure has yielded satisfactory results. However, bleeding at the distal anastomosis of the aortic arch is a main risk of this type of operation. The aortic wall lesion receives limited surgical exposure and is thin, fragile, and easily torn. Moreover, once distal anastomotic bleeding occurs, it is difficult to perform hemostasis, and internal drainage may be ineffective. Although the stent-in-stent procedure supports the suture and can significantly reduce the incidence of anastomotic bleeding, it also has some inevitable shortcomings, as follows. (1) stenosis of the lumen, especially in patients with primitive lumen stenosis, results in reduced effective blood flow and ischemia of the lower half of the body. (2) Thrombosis at the distal end of elephant trunk stent causes embolism. (3) Distortion, folding, and collapse of the stent vessels can lead to hemolysis and distal dysuria.

To address the limitations of the stent-in-stent procedure, we made a technical improvement based on a graft inversion technique which was first applied to distal anastomosis in a total arch replacement for thoracic aortic aneurysm by Koyu Tanaka et al. [3]. We first applied this improvement to the treatment of RTAD. Our experience has demonstrated the advantage of our graft inversion technique for the treatment of this serious complication. This technique simplifies the operative procedure while allowing anastomosis to be performed under good surgical exposure. Our improved procedure also reduces the duration of CPB time, and circulatory arrest time and does not require graft-to-graft anastomosis. Moreover, the “sandwich” suture is firm and can be used to compress the aortic wall after the inverted graft is pulled out, thus reducing the risk of anastomotic bleeding. Finally, our procedure avoids the previous issue involving the difficulty of suturing endovascular stent grafts to vascular grafts. We further note that with this procedure, there are no reduction in the vessel lumen, compared with elephant trunk stent implantation, and no elephant trunk stent-related complications.

### Limitations

There are some inevitable limitations of the present study that should not be neglected. First of all, this is a retrospective study, the completeness of related clinical data may have affected the results. Secondly, this is a review of a single-center experience. Surely there are disparities among different institutions and operators. Finally, as the small sample size, the short follow-up period, long-term clinical trials with large sample are needed to confirm the feasibility and safety of graft Inversion technique and surgical intervention for RTAD after TEVAR.

## Conclusions

Awareness of RTAD should be heightened after TEVAR. The graft inversion anastomosis technique can enable a secure distal anastomosis under good surgical exposure, resulting in reduced durations of CPB, and circulatory arrest for RTAD after TEVAR. Surgical treatment could be is a safe alternative for treatment of this patients.

## Additional files

Additional file 1: **Figure S1.** Diagram of the key steps of graft inversion technique. (A) After the inverted graft was inserted into the descending aorta, distal anastomosis was performed under a good surgical exposure. (B) The inverted graft is pulled up proximally after distal anastomosis was completed. (PNG 12362 kb)

Additional file 2: A video of graft inversion technique. This additional video file shows the graft inversion technique in more detail. (MP4 116134 kb)

## Abbreviations

AVP: Aortic valve repair
AVR: Aortic valve replacement
CPB: Cardiopulmonary bypass
CTA: Computed tomography angiography
RTAD: Retrograde type A aortic dissection
SCP: Selective cerebral perfusion
SET: Stented elephant trunk
TBAD: Type B aortic dissection
TEVAR: Thoracic endovascular aortic repair

## References

[1] Dake MD, Kato N, Mitchell RS (1999). Endovascular stent-graft placement for the treatment of acute aortic dissection. N Engl J Med.

[2] Nienaber CA, Fattori R, Lund G (1999). Nonsurgical reconstruction of thoracic aortic dissection. N Engl J Med.

[3] Tanaka K, Yoshitaka H, Irie Y (2012). Branched graft inversion technique for distal anastomosis in total arch replacement. Ann Thorac Surg.

[4] Laquian L, Scali ST, Beaver TM (2018). Outcomes of thoracic endovascular aortic repair for acute type B dissection in patients with intractable pain or refractory hypertension. J Endovasc Ther.

[5] Preventza O, Garcia A, Moeller K (2015). Retrograde ascending aortic dissection after thoracic endovascular aortic repair for distal aortic dissection or with zone 0 landing: association, risk factors, and true incidence. Ann Thorac Surg.

[6] Williams JB, Andersen ND, Bhattacharya SD (2012). Retrograde ascending aortic dissection as an early complication of thoracic endovascular aortic repair. J Vasc Surg.

[7] Preventza O, Garcia A, Moeller K (2015). Retrograde ascending aortic dissection after TEVAR for distal aortic dissection or with zone 0 landing. Association, risk factors, and true incidence. Ann Thorac Surg.

[8] Chen Y, Zhang S, Liu L (2017). Retrograde type a aortic dissection after thoracic endovascular aortic repair: a systematic review and meta-analysis. J Am Heart Assoc.

[9] Kpodonu J, Preventza O, Ramaiah VG (2008). Retrograde type a dissection after endovascular stenting of the descending thoracic aorta. Is the risk real?. Eur J Cardiothorac Surg.

[10] Van Prehn J, Schosser FJ, Muhs BE (2009). Oversizing of aortic stent grafts for abdominal aneurysm repair:a systematic review of the benefits and risks. Eur J Vasc Endovasc Surg.

[11] Dong ZH, Fu WG, Wang YQ (2009). Retrograde type a aortic dissection after endovascular stent graft placement for treatment of type B dissection. Circulation.

[12] García Reyes ME, Gonçalves Martins G, Fernández Valenzuela V, et al. Long-term outcomes of thoracic endovascular aortic repair focused on bird beak and oversizing in blunt traumatic thoracic aortic injury. Ann Vasc Surg. 2018;50:140–7. 10.1016/j.avsg.2018.02.001 29455010

[13] Canaud L, Ozdemir BA, Patterson BO, et al. Retrograde aortic dissection after thoracic endovascular aortic repair. Ann Surg. 2014;260:389–95. 10.1097/SLA.0000000000000585 24441822

[14] Liu L, Zhang S, Lu Q (2016). Impact of oversizing on the risk of retrograde dissection after TEVAR for acute and chronic type B dissection. J Endovasc Ther.

[15] Spiliotopoulos K, Preventza O, Green SY (2018). Open descending thoracic or thoracoabdominal aortic approaches for complications of endovascular aortic procedures: 19-year experience. J Thorac Cardiovasc Surg.

[16] Eggebrecht H, Thompson M, Rousseau H (2009). Retrograde ascending aortic dissection during or after thoracic aortic stent graft placement: insight form the European registry on endovascular aortic repair complications. Circulation.

[17] Tjaden BL, Sandhu H, Miller C (2018). Outcomes from the Gore global registry for endovascular aortic treatment in patients undergoing thoracic endovascular aortic repair for type B dissection. J Vasc Surg.

